# Direct fluorescence detection of VirE2 secretion by *Agrobacterium tumefaciens*

**DOI:** 10.1371/journal.pone.0175273

**Published:** 2017-04-12

**Authors:** Noga Yaakov, Yoav Barak, Idan Pereman, Peter J. Christie, Michael Elbaum

**Affiliations:** 1 Dept of Materials and Interfaces, Weizmann Institute of Science, Rehovot, Israel; 2 Chemical Research Support Dept, Weizmann Institute of Science, Rehovot, Israel; 3 Dept of Plant and Environmental Sciences, Weizmann Institute of Science, Rehovot, Israel; 4 Department of Microbiology and Molecular Genetics, UT-Houston Medical School, Houston, Texas, United States of America; Centre National de la Recherche Scientifique, Aix-Marseille Université, FRANCE

## Abstract

VirE2 is a ssDNA binding protein essential for virulence in *Agrobacterium tumefaciens*. A tetracysteine mutant (VirE2-TC) was prepared for *in vitro* and *in vivo* fluorescence imaging based on the ReAsH reagent. VirE2-TC was found to be biochemically active as it binds both ssDNA and the acidic secretion chaperone VirE1. It was also biologically functional in complementing *virE2* null strains transforming *Arabidopsis thaliana* roots and *Nicotiana tabacum* leaves. *In vitro* experiments demonstrated a two-color fluorescent complex using VirE2-TC/ReAsH and Alexa Fluor 488 labeled ssDNA. *In vivo*, fluorescent VirE2-TC/ReAsH was detected in bacteria and in plant cells at time frames relevant to transformation.

## Importance

Cell to cell transfer of proteins and nucleic acids lies at the heart of many biological processes. Detecting such transfer is often problematic or indirect. We show here that the biarsenical fluorescent reagent ReAsH can be used to reveal the presence of the secreted effector VirE2 from *Agrobacterium tumefaciens* in the cytoplasm of host plant cells. Our studies establish a new method for monitoring translocation of the VirE2 effector from *A*. *tumefaciens* to plant target cells during the infection process.

## Introduction

*Agrobacterium tumefaciens* is a Gram negative soil pathogen of plants. It has the ability to transform dicotyledonous plants, leading to formation of tumors known as “crown galls”. This disease results from the transfer of a single strand DNA segment from the large tumor inducing (Ti) plasmid carried by the bacterium into the plant host cell [1]. Based on a type IV secretion system, the transfer process is similar to bacterial conjugation. Virulence genes (*vir*) responsible for the DNA transfer reside on the same Ti-plasmid as the transferred DNA (T-DNA). These encode for protein components of the secretion system itself, for the processing machinery to excise a single strand of T-DNA and bind a mobility protein (VirD2) to the 5’ end, and for accompanying effector proteins [2]. The most abundant of these is VirE2, a 63.5 kDa ssDNA-binding protein that is essential for efficient transformation [3–5]. In vitro, VirE2 binds the T-strand cooperatively, without sequence specificity. The resulting T-complex is a solenoidal structure that has been extensively characterized [6–9]. In planta, VirE2 protects the T-strand from degradation [10], though evidence for a direct interaction is still lacking. VirE2 possesses nuclear localization signals [11–13] and interacts with a specific importin-α isoform, IMP-α4 [14], as well as with the host plant transcription factor VIP1 [15,16].

In evidence for an interaction in the host cell, the T-strand and VirE2 protein may be transported to it separately. Extracellular complementation assays showed that coinoculation of plant with a combination of two *Agrobacterium* strains, one lacking T-DNA but containing VirE2, the other lacking VirE2 but containing T-DNA, leads to successful plant transformation [17]. Moreover, VirE2 expressed exogenously in plant cells binds T-strand delivered from *A*. *tumefaciens* to mediate formation of tumors, even if the infecting bacterial strain lacks VirE2 [11]. Nonetheless the parameters governing encounter between VirE2 and the T-strand remain unknown when these do originate, as naturally, from the same bacterium.

In *Agrobacterium tumefaciens*, VirE2 is expressed together with VirE1, a small acidic chaperone that is essential for VirE2 secretion and pathogenicity [18,19]. VirE2 expressed *in vitro* in the absence of VirE1 is prone to aggregation and forms disordered filamentous structures [8]. This may represent the biologically active form if the protein is secreted independently of the T-strand and without VirE1. In the presence of ssDNA, VirE2 adopts a more ordered solenoidal form with N to C terminal contacts azimuthally around a hollow core [6,7,9]. The structure of the VirE1-VirE2 complex was investigated by X-ray crystallography [20]. The protein contains two major folded domains of similar structure, joined by a flexible linker. The two domains clamp tightly a single alpha helix in VirE1, constraining the termini to an orientation incompatible with oligomerization.

In the plant cell the T-DNA is thought to be directed to the nucleus by VirE2 as well as nuclear localization signals (NLS) on VirD2 [12,21]. T-complex labeling *in vitro* was described in the literature, producing 'artificial' fluorescent T-complex. In one study, VirE2 was bound to fluorescently labeled ssDNA to produce the T-complex *in vitro*, which was then microinjected into *Tradescantia* stamen hair cells. These complexes reached the nucleus [22], while fluorescent ssDNA injected independently of VirE2 remained cytoplasmic. Other evidence indicate a cytoplasmic localization of VirE2, however [14,23], motivating further examination by direct imaging.

*In vivo* visualization of VirE2 was reported recently. The approach taken was based on split green fluorescent protein, either in a bimolecular fluorescence complementation (BiFC) mode or by opening the beta barrel at sheet 11 [24–26] following Cabantous [27]. VirE2 was found in both cytoplasm and nucleus of *Nicotiana benthamiana* cells [24,26], but only in the cytoplasm of *Saccharomyces cerevisiae* [24,25]. However, these studies detected VirE2 not earlier than one or more days post-infection by the bacterium. Transcription of the transgenes occurs with as little as two hours co-inoculation [28], implying delivery of both T-DNA and VirE2 on that time scale.

Here we present an alternative approach to labeling of VirE2 *in vitro and in vivo* by introducing a tetracysteine (TC) motif to a flexible loop in the protein. The TC motif is recognized by a non-fluorescent biarsenical derivative of Resorufin, known as ReAsH [29]. A similar approach was employed to follow *Shigella flexneri* effector secretion to human tissue culture cells using the related reagent FlAsH [30]. We show that the VirE2-TC protein displays the expected biochemical interactions and is biologically functional for transformation. We detected it abundantly in live bacteria, and at low abundance in infected plant tissue.

## Materials and methods

### Bacterial strains and growth conditions

*E*. *coli* DH5α and BL21 bacteria were grown in LB medium [31], overnight at 37°C in a shaker (250 rpm). *Agrobacterium* strains are listed in Table 1. *Agrobacterium* were grown in YEP [32] antibiotic (kancmycin 50 μg/ml, carbenicillin 100 μg/ml spectinomycin 250 μg/ml streptomycin 250 μg/ml) overnight at 28°C, in a shaker (250 rpm). For VirE2-TC expression in the AT12516 (*virE2*- mutant) strain, bacteria were grown overnight in YEP medium at 28°C, in a shaker (250 rpm). Cells were then diluted 1:25 in AB minimal medium [33] + 0.5% glucose and grown until OD_600_ = 0.6, then cells were diluted to OD_600_ = 0.2 in AB induction medium (AB + 0.5% glucose +200 μM acetosyringone +50 mM MES pH 5.5) overnight at 20°C in a shaker (250 rpm).

**Table 1:** Agrobacterium strains.

Strain	Description	Reference
EHA105; ΔT-DNA	EHA101 derivative	[52]
EHA105 (pCAMBIA2301)	Binary plasmid carries T-DNA encoding a beta-glucuronidase (GUS) reporter gene. (Kan^r^)	http://www.cambia.org/daisy/cambia/585
EHA105 (pCAMBIA2300-GFP)	Binary plasmid carries T-DNA encoding a GFP reporter gene (Kan^r^)	Prof. Yedidia Gafni, Agricultural Research Organization, Volcani Center
AT12516	*virE2* null strain (Spec^r^, Strep^r^ and Carb^r^)	[36]
AT12516(pCAMBIA2301)	*virE2* null strain with binary plasmid encoding beta-glucuronidase (GUS) reporter gene. (Spec^r^, strep^r^ carb^r^ and kan^r^)	This study
AT12516(pCAMBIA 2301-VirE2)	*virE2* null strain with binary plasmid encoding beta-glucuronidase (GUS) reporter gene expressed from the CaMV35S promoter and VirE2 expressed from the *virB* promoter. (Spec^r^, strep^r^ carb^r^ and kan^r^)	This study
AT12516(pCAMBIA 2301:VirE2-TC)	*virE2* null strain with binary plasmid carries T-DNA encoding a Beta-glucuronidase (GUS) reporter gene expressed from the CaMV35S promoter and VirE2-TC expressed from the *virB* promoter. (Spec^r^, strep^r^ carb^r^ and kan^r^)	This study
PC 1000	A348 strain lacking the *virB* operon	[53]

#### Cloning of wild type *virE2* and *virE2-TC* genes

All PCR reactions were performed using high fidelity DNA polymerase, Pwo (Roche) for small DNA fragments, and Phusion (FINNZYMES) for larger ones, using primers reported in Table 2. For *in vitro* experiments, wild-type nopaline *virE2* was cloned into pHIS Parallel1 plasmid [34] in Nco1 and Sal1 restriction sites with 6X-His tag in *E*.*coli* DH5α strain. For *virE2-TC* cloning, the TC motif was inserted in *virE2* gene according to Adams [35], **Cys-Cys-Pro-Gly-Cys-Cys**, using two steps of PCR.

**Table 2:** List of primers.

Name	Sequence	uses
NCOI F	5'–GGC GCC ATG GAT CCG AAG GCC GAA GGC AAT- 3'	Cloning of *vvirE2* into pHIS vector
SAL R	5'–CTC GTC GAC GCT ACA GAC TGT TTA CGG TTG- 3'	Cloning of *vvirE2* into pHIS vector
VIRE2 mut F	5'- TGC TGT CCA GGC TGC TGT CAG TTT CCG GCT GCG ACT GT- 3'	Introduction of tetracysteine codons into *virE2*
VIRE2 mut R	5'- ACA GCA GCC TGG ACA GCA CTC GCC GGC GAA CTC TGC- 3'	Introduction of tetracysteine codons into *virE2virE2*
Nde1 F	5'- GGC CAT ATG GAT CCG AAG GCC GAA GGC AAT- 3'	Cloning of *virE2-TC* into pACYDuet-1:*virE1* vector
Kpn1 R	5'- GTC GGT ACC CTA CAG ACT GTT TAC GGT TGG- 3'	Cloning of *virE2-TC* into pACYDuet-1:*virE1* vector
virB F	5'- TCG CCC GGG ACA GGC TTA TGT CCA- 3'	Cloning of *virB* promoter+*virE2* or *virET-TC* into pCAMBIA2301 vector
virB R	5'- CGA CCC GGG CTA CAG ACT GTT TAC- 3'	Cloning of *virB* promoter+*virE2* or *virE2-TC* into pCAMBIA2301 vector

For *in vivo* experiments, the *virE2-TC* gene under the *virB* promoter was cloned into a binary plasmid pCAMBIA2301 for expression in the bacterium (i.e., not in the T-DNA). The nopaline *virB* promoter sequence was taken from pDW029 plasmid [36]. The *virE2* gene with *virB* promoter was cloned into the SgrAI restriction site in order to synchronize its expression with the other virulence genes. Wild-type VirE2 was cloned similarly. pCAMBIA2301:VirE2-TC and pCAMBIA2301:VirE2 were transformed into the VirE2 null strain AT12516.

### Expression and purification of wild type VirE2 and VirE2-TC proteins

Recombinant proteins were purified from inclusion bodies under denaturing conditions in buffer containing 6M guanidine hydrochloride, in order to prevent aggregation and precipitation. The wild type *virE2* and *virE2-TC* nopaline genes were cloned in pHIS parallel1 plasmid [34] with 6x-His tags between the restriction sites Nco1 and Sal1 and the resulting plasmids were introduced into BL21 (DE3) for protein production. Five ml overnight cultures were inoculated into 500 ml LB and resulting cultures were grown to an OD_600_ = 0.6. Isopropyl β-D-1-thiogalactopyranoside (IPTG) was added at a final concentration of 0.1 mM, and resulting cultures were incubated at 37°C for 3h to induce synthesis of VirE2 or at 30°C for 5h for VirE2-TC production. Cells were harvested, suspended in buffer A (20 mM sodium phosphate, 500 mM NaCl, pH 7.4) and sonicated. Inclusion bodies were collected by centrifugation, washed twice with buffer A, and denatured by suspension in buffer A + 6M guanidine hydrochloride and 20 mM imidazole for 30 min at room temperature. The denatured proteins were loaded on His-Trap HP 1ml column (GE healthcare) and purified using the AKTA-basic system (GE healthcare). The column was washed with 10 column volumes (CV) of buffer A + 20 mM imidazole and bound proteins were eluted using a linear gradient (20 mM to 500 mM) of imidazole in buffer A + 6 M guanidine hydrochloride. Fractions were collected and refolded by dialysis against 2 L of buffer B (100 mM NaCl, 40 mM sodium phosphate, 10% glycerol, 1 mM DTT, pH 7.4) and analyzed by SDS-PAGE.

### VirE1-VirE2-TC co-expression and purification

VirE2-TC was cloned into pACYCDuet-1:VirE1 (obtained from the Israel Structural Proteomics Center, Weizmann Institute of Science). VirE1 was expressed with 6x-His tag while VirE2-TC was untagged. The plasmid was transformed into *E*.*coli* BL21 (DE3) strain for protein synthesis. Cells were grown overnight, diluted in the morning 1:100 into 500 ml LB, and regrown until OD_600_ = 0.6. Cells were induced overnight at 16°C by adding Isopropyl β-D-1-thiogalactopyranoside (IPTG) to a final concentration of 0.1 mM. Cells were harvested and suspended with 20 ml of buffer A and protease inhibitor cocktail (Sigma). The suspended cells were sonicated and centrifuged, the supernatant was loaded on a His-Trap HP 1ml column (GE Healthcare), and soluble proteins were purified using AKTA-basic system (GE Healthcare). The column was washed with 10 CV of buffer A + 20 mM imidazole, and bound His-tagged proteins were eluted from the column using a linear gradient (20 mM to 500 Mm) of imidazole in buffer A. Fractions were collected and refolded by dialysis against 2 L of buffer B and analyzed by SDS-PAGE.

### Electrophoretic gel mobility shift assay of protein-ssDNA binding

Purified VirE2 and VirE2-TC proteins were mixed with M13 ssDNA in binding buffer as described below. The mixing ratios (w/w) ssDNA:protein were 1:1, 1:5,1:10 and 1:15. Samples were incubated overnight at 4°C for DNA-protein complex formation, and then analyzed by electrophoresis through an 0.8% agarose gel and ethidium bromide staining. The stained gel was scanned in FUJIFILIM FLA-5100 using a green laser (543 nm) and analyzed by Multi Gauge software.

### Transmission electron microscopy analysis of VirE2-ssDNA binding

M13mp18 single strand DNA (New England Bio-labs) was denatured by heating at 70°C for five min and then cooled on ice for five min. Wild-type VirE2 and VirE2-TC proteins were added to the ssDNA in binding buffer (10 mM Hepes pH 7.4, 150 mM NaCl, 1 mM EDTA, 1 mM DTT) at a ratio of 1:10 (w/w) ssDNA:protein for full coverage of the DNA. The ssDNA-protein samples were incubated for 3h or overnight at 4°C to form the complex. For TEM analysis, 5 μl of the ssDNA-protein complex was placed on glow discharged carbon coated copper grids and negatively stained with 1% uranyl acetate for 50 sec. Samples were imaged in a Tecnai Spirit BioTWIN (FEI) operating at 120 kV. Images were recorded with an Eagle 2K*2K CCD camera (FEI).

### *In vitro* labeling of VirE2-TC

The VirE2-TC protein was labeled *in vitro* by ReAsH reagent (obtained from Invitrogen as Lumio Red In-Cell labeling kit, cat no 12589–040). M13 ssDNA was first labeled with Alexa Fluor 488 dye (ULYSIS nucleic acid labeling kit, Molecular Probes) and then mixed with purified proteins, VirE2-TC and VirE2 as a control at room temperature for 3h or overnight at 4°C. The assembled complexes were labeled with ReAsH reagent at a final concentration of 100 nM. These reactions were incubated for 3 h in darkness to produce fluorescent VirE2-TC. For imaging by laser scanning confocal microscopy (Olympus Fluoview 300 with 60x/1.4NA oil-immersion objective), the complexes were mixed with 3% agarose to yield a final concentration of 1% agarose and hardened quickly into a gel inside the sample chamber. Samples were excited at two wavelengths,488 nm for Alexa Fluor 488 and 543 nm for ReAsH. Fluorescence emission was recorded at 510–530 nm for Alexa Fluor 488-DNA and at 600–620 nm for the ReAsH-VirE2-TC.

### *In vivo* labeling of VirE2-TC in *Agrobacterium*

For *in vivo* labeling of VirE2-TC protein inside the bacteria, the *virE2*^-^ *Agrobacterium* strain AT12516 [37] carrying the *virE2-TC* gene was induced in minimal medium (see growth conditions). The induced cells were washed once with 50 mM HEPES (pH 8) and treated with 650 μM BAL (2,3-dimercaptopropanol) in 50 mM HEPES for 15 min to prevent the nonspecific binding of ReAsH reagent. Then cells were washed with 50 mM HEPES, and 0.5–2 μM of ReAsH reagent was added at the final OD_600_ 0.5. Cells were incubated for 3 h at room temperature in darkness and then washed three times with 250 μM BAL and once with 250 μM BAL plus 20 μM Disperse Blue 3 (obtained from Invitrogen) for removal of excess reagent. Cell samples (5 μl) were placed onto a glass slide and analyzed by laser scanning confocal microscopy using 543 nm excitation and emission detection at 600–620 nm.

### *In vivo* labeling of VirE2-TC in plant cells

*Agrobacterium* strain AT12516 carrying the *virE2-TC* gene was induced in LB- MES media (see plant transformation methods). Cells were infiltrated to *Nicotiana tabacum* and *Nicotiana benthamiana* leaves. At 4 to 20 hours post- infection, hand cut tissues were subjected to ReAsH labeling. Tissues were pretreated with 650 μM BAL for 15 min to reduce the nonspecific binding of the reagent, rinsed, and incubated for 30–45 min with 1–2 μM ReAsH in water in darkness. Treated tissues were washed three times with 250 μM BAL and once with 250 μM BAL plus 20 μM Disperse Blue 3. Labeled tissues were analyzed by laser scanning confocal microscopy (Olympus Fluoview 300) using a 60x/1.2NA water-immersion objective. Fluorescence and transmitted light differential interference contrast images were collected simultaneously with a spatial sampling of 90 nm/pixel.

### 3D image processing and intensity analysis

Three dimensional (XYZ) recordings were analyzed offline by the following procedure. Image stacks were first projected (as maximum intensity) down the Z axis in order to find all the fluorescent spots. The slices generating each individual spot were then cropped and inspected visually, together with the differential interference contrast channel that was acquired simultaneously. Fluorescent particles smaller than the diffraction limit of the microscope display the characteristic size and shape of the 3D confocal point spread function. The total spot intensities, on the other hand, reflect the density of fluorophores within the diffraction-limited spot. Therefore projected intensities in the cropped spots (as integrated sum) were tabulated for each detected particle as a measure of the quantity of ReAsH-labeled VirE2. Images were analyzed by Fiji [38] and plots prepared in Origin software (OriginLab Corp).

### Plant transformation methods

Agroinfiltration assay: *Nicotiana tabacum* and *Nicotiana benthamiana* leaves. Overnight cultures of *Agrobacterium* diluted 1:20 into LB-MES buffer pH 5.9 with 20 μM acetosyringone and resulting cultures were incubated to a final OD_600_ = 0.5–0.6. Cells were pelleted and resuspended in a solution containing: 10 mM MgCl_2_, 10 mM MES, and 150 μM acetosyringone, incubated at room temperature for 2–3 h and infiltrated with a 1 ml syringe into the leaves.

*Arabidopsis thaliana* root transformation: Transient expression of Beta-glucuronidases- GUS in *Arabidopsis* roots was detected by treatment with GUS substrate 5-bromo-4-chloro-3-indolyl glucuronide (x-gluc) as previously described [39].

Complementation assay: Functionality of VirE2-TC in mediating successful transfer of T-DNA to plants was assessed by extracellular complementation as follows. Two AT12516 (*virE2*^-^) strains, one carrying a T-DNA binary vector encoding the GFP protein and a second encoding either VirE2 or VirE2-TC, were induced with acetosyringone. The cultures were mixed and infiltrated into *Nicotiana tabacum* plant leaves. Successful transfer of T-DNA encoding GFP by the first bacterial strain to the plant nucleus requires co-transfer of a functional form of VirE2 (or VirE2-TC) by the second bacterial strain into the same plant cell. To confirm co-transfer of T-DNA and functional VirE2 (or VirE2-TC), plant cells were monitored for GFP production monitored after 48 h of infection by laser scanning confocal microscopy.

## Results

### Molecular interactions of VirE2-TC *in vitro*

**Fig 1:**
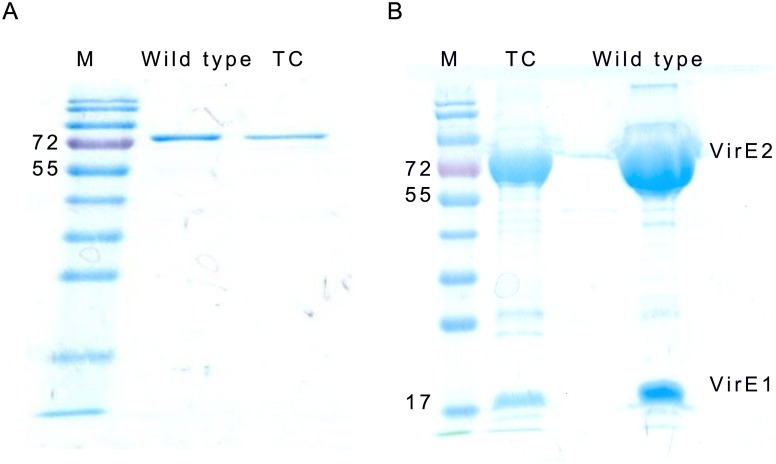
Expression of TC-modified VirE2 mutant. (A) wtVirE2 and VirE2-TC purified from inclusion bodies co-migrate in SDS-PAGE analysis. (B) Co-expression of 6xHis-tagged VirE1 with wtVirE2 or VirE2-TC led to a soluble product containing both proteins.

We introduced tetracysteine (TC) motif **Cys-Cys**-Pro-Gly-**Cys-Cys** replacing amino acids 339 to 344 of VirE2 to generate VirE2-TC. This region represents an unstructured linker between the two major folded domains of the protein, as shown by the VirE1-VirE2 crystal structure [20]. We first analyzed biochemical properties of VirE2 and VirE2-TC in vitro by addition of 6x-His tags and purification by immobilized metal affinity chromatography (IMAC). In the absence of its small acidic chaperone VirE1, VirE2 has a strong tendency to oligomerize into poorly soluble, irregular filaments [8]. Therefore purification was performed from inclusion bodies. VirE2 and VirE2-TC co-migrated in SDS-polyacrylamide gels, although VirE2-TC purified at lower concentrations than VirE2 (Fig 1A).

### VirE1-VirE2-TC interaction in co-expression

We tested for an interaction between VirE2-TC and VirE1 by coproducing VirE2-TC and and 6xHis tagged VirE1 in the same cell following Dym et al [20]. Due to high solubility of the heterodimer protein, purification was performed under native conditions. VirE1 interacted similarly with wild-type VirE2 and VirE2-TC (Fig 1B). The acidic VirE1 runs in gel electrophoresis with a misleadingly large apparent molecular weight. MALDI mass spectrometry analysis confirmed that the lower bands in Fig 1B are indeed VirE1 (data not shown).

### ssDNA–VirE2 and ssDNA–VirE2–TC interaction assays

**Fig 2:**
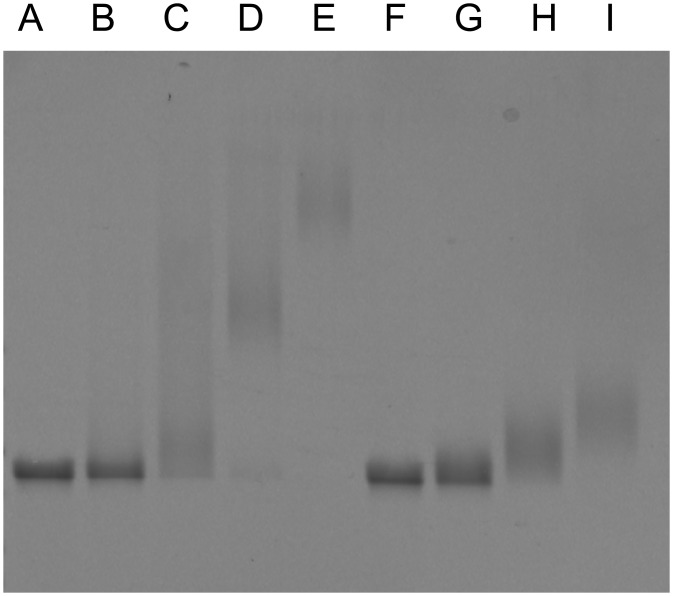
Electrophoretic Mobility Shift Analysis (EMSA) confirms binding of VirE2 to M13 ssDNA substrate. M13 ssDNA concentration was held constant, while relative protein concentration increased. Lanes: (A) M13 alone, B-E) wtVirE2 at 1:1, 1:5, 1:10, 1:15 ratios wt:wt, (F-I) VirE2-TC at equal ratios. VirE2-TC binds M13 ssDNA less avidly than the wild-type, but leaves no evidence of completely unbound ssDNA.

**Fig 3:**
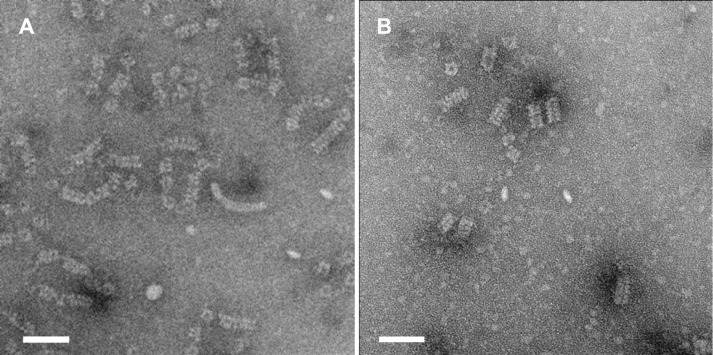
Transmission electron microscopy confirms the formation of VirE2-DNA complexes with similar structure. (A) wild-type and (B) TC-mutant VirE2. Scale bar 50 nm.

Next, we tested for ssDNA binding activity of VirE2-TC by electrophoretic mobility shift assay (EMSA) and transmission electron microscopy (TEM). For EMSA, VirE2 and VirE2-TC were mixed at a series of increasing protein concentrations with M13 circular ssDNA. Both proteins shifted the ssDNA species in agarose gels, although VirE2-TC was less efficient in ssDNA-binding than the native protein (Fig 2). When analyzed by TEM, we detected full coverage of VirE2 bound along the M13 ssDNA substrate (Fig 3A). VirE2-TC, however, bound in shorter segments along the substrate and a considerable amount of protein was detected in the background (Fig 3B). Thus, VirE2-TC binds single-stranded DNA, albeit less efficiently than VirE2, and also forms solenoidal capsids characteristic of the native protein.

### Biological function of VirE2-TC in plants

**Fig 4:**
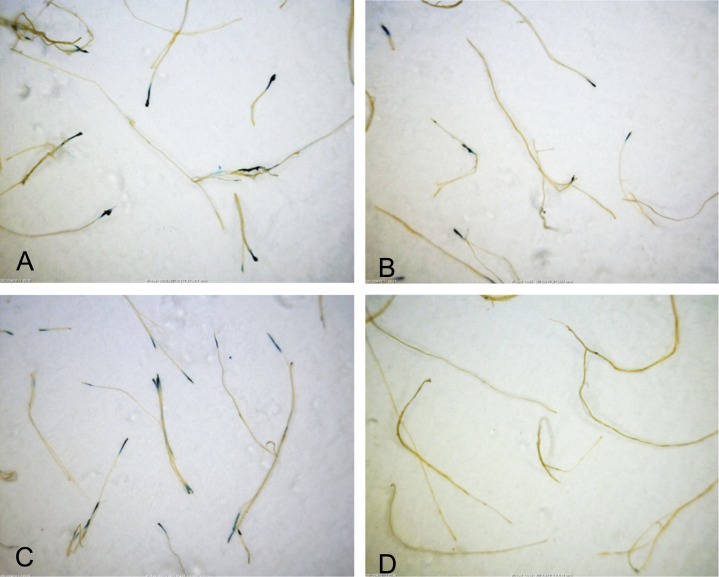
GUS expression in *Arabidopsis* roots. (A) Infection by VirE2-null strain with both wild-type VirE2 expression and GUS gene transfer from binary plasmid pCAMBIA2301. (B) Infection by VirE2-null strain with both VirE2-TC expression and GUS gene transfer from binary plasmid pCAMBIA2301. (C) Positive control: GUS expression in roots infected by wild type strain transferring GUS gene from pCAMBIA2301 vector. (D) Negative control: roots infected by VirE2-null strain carrying the same GUS gene on pCAMBIA2301 vector.

The biological function of VirE2-TC was assayed in *Nicotiana tabacum* leaves and in *Arabidopsis* roots. Two strategies were employed based on binary plasmids introduced into the *virE2* mutant strain AT12516. First, we monitored transfer of T-DNA bearing a GUS reporter gene by assaying for GUS activity in infected *Arabidopsis* roots. The infecting bacterium carried the GUS T-DNA binary vector pCAMBIA2301 that was engineered to express either VirE2-TC or native wtVirE2 in the bacterium, from the *virB* promoter. Roots infected with either strain exhibited similar blue staining indicative of successful delivery of the GUS reporter gene and its expression in the plant cells (Fig 4A and 4B). In control experiments, wild-type *A*. *tumefaciens* carrying pCAMBIA2301 elicited GUS report activity in plants upon infection (Fig 4C), whereas *A*. *tumefaciens* AT12516 carrying pCAMBIA2301 but lacking genes for VirE2 or VirE2-TC failed to elicit GUS reporter activity (Fig 4D).

**Fig 5:**
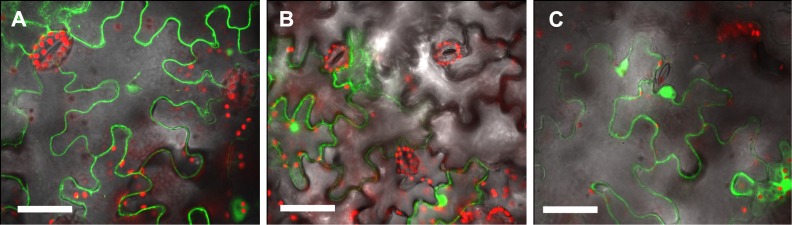
Complementation assay in *Nicotiana tabacum* leaves. (A) Positive control: GFP expression in leaf infiltrated with wild-type *Agrobacterium* carrying binary plasmid pCAMBIA2300-GFP. (B) Complementation by VirE2-TC: GFP expression in leaf tissue infiltrated with two VirE2-null *Agrobacterium* strains (AT12516), one carrying pCAMBIA2300-GFP and the other expressing VirE2-TC from a binary plasmid pCAMBIA2301:VirE2-TC. (C) Complementation by wild-type VirE2: GFP expression in leaf infiltrated with two VirE2-null *Agrobacterium* strains (AT12516), one carrying pCAMBIA2300-GFP and the other expressing wtVirE2 from binary plasmid pCAMBIA2301:VirE2. autofluorescence of chlorophyll in red. Scale bar 50 μm.

Next, we monitored transfer of T-DNA bearing a GFP reporter gene to *Nicotiana tabacum* leaves by mixed infection. In this assay, plant tissue is inoculated with a mixture of two strains, one proficient for transfer of T-DNA (encoding GFP for expression in the plant) and the second for transfer of VirE2 or VirE2-TC protein. If the two strains deliver their respective substrates into the same plant cell, complex formation in the plant results in successful delivery of the T-DNA to the plant nucleus and synthesis of the GFP reporter. Fig 5 demonstrates biological function of VirE2-TC in gene transfer. Three bacterial strains were prepared in the *virE2* null background AT12516: one containing a pCAMBIA binary plasmid carrying only the T-DNA(GFP) sequence, one expressing VirE2-TC for secretion, and expressing wtVirE2 for secretion. Additionally, the EHA105 strain (expressing wtVirE2) was transformed with T-DNA(GFP) as a positive control. This positive control (Fig 5A) shows the distribution of expressed GFP delivered in a single infection. Mixed infection by a strain carrying only T-DNA with a second strain expressing either VirE2-TC (Fig 5B) or wtVirE2 (Fig 5C) showed similar GFP expression patterns. In control experiments, infections with the strain carrying T-DNA(GFP) alone failed to incite GFP production (data not shown).

Taken together, results of the *Arabidopsis* root and *N*. *tabacum* leaf transformation studies establish that *A*. *tumefaciens* efficiently delivers VirE2-TC to plant cells and, furthermore, that VirE2-TC exhibits WT protein function in mediating T-DNA transfer to the plant nucleus.

### *In vitro* labeling of T-complex

**Fig 6:**
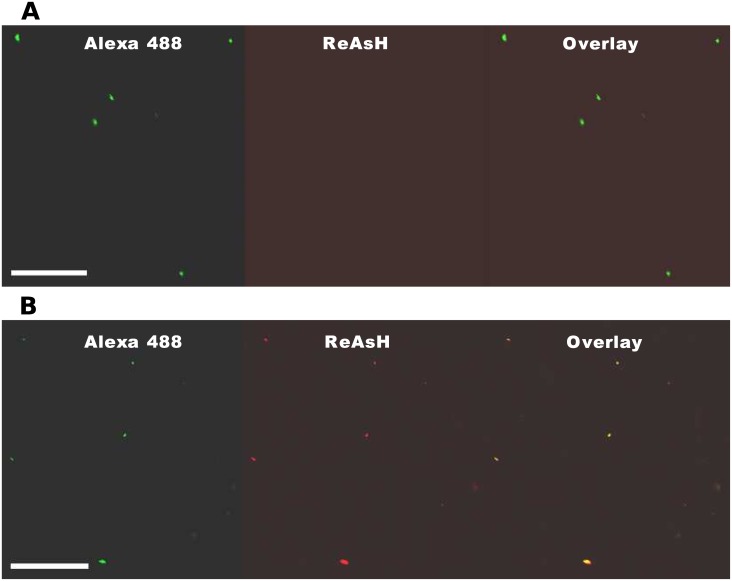
Confocal microscopy images of ssDNA-protein particles formed by M13 circular ssDNA labeled with Alexa 488. (A) wild type VirE2, (B) VirE2-TC. Scale bar 50 μm.

Having shown that VirE2-TC interacts with VirE1 and binds ssDNA *in vitro*, and displays biological function in plant transformation assays, we next tested for labeling of the tetracysteine motif with the ReAsH reagent. ReAsH binding to VirE2-TC protein was assayed with complexes formed on M13 ssDNA pre-labeled with Alexa Fluor 488 dye (See Materials and methods). Complexes formed with native VirE2 on fluorescent DNA served as a negative control against non-specific binding of the ReAsH. As shown in Fig 6A, complexes composed of native VirE2 and DNA exhibited fluorescence of the labeled DNA substrate, but no detectable ReAsH fluorescent signal. By contrast, complexes composed of VirE2-TC and DNA, exhibited fluorescent VirE2-TC spots that colocalized with the labeled DNA substrate when analyzed by laser scanning confocal microscopy (Fig 6B).

### *In vivo* labeling of VirE2-TC protein in *Agrobacterium*

**Fig 7:**
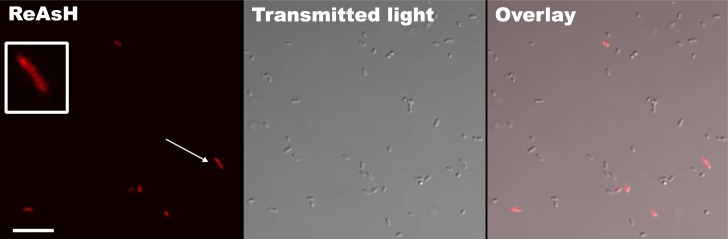
*In vivo* labeling of VirE2-TC. ReAsH labeling of virulence activated bacteria expressing VirE2-TC. Inset: zoom of a single fluorescent bacterium (white arrow) shows polar accumulation of the fluorescent label. Scale bar 10 μm.

We assayed for in vivo binding of the ReAsH reagent to VirE2-TC in *A*. *tumefaciens* as follows. AT12516 carrying pCAMBIA2301 with P_*virB*_::*virE2-TC* was treated with acetosyringone to induce synthesis of VirE2-TC. We then treated cells with BAL (2,3-dimercaptopropanol), a reducing agent shown previously to prevent nonspecific cysteine labeling by the ReAsH reagent [40]. Following a wash step, cells were treated with the ReAsH reagent, which is membrane permeable and thus can enter the cell without permeabilization. As shown in Fig 7, approximately 5–15% of the treated cells were fluorescent, whereas similarly treated isogenic cells producing native VirE2 remained dark (data not shown). Thus, the VirE2-TC protein is abundantly labeled with the fluorescent reporter in induced *A*. *tumefaciens* cells.

### *In vivo* labeling of VirE2-TC in plants

Our ultimate aim was to visualize transferred VirE2 in plant cells *in vivo*. To this end, *Nicotiana tabacum* and *Nicotiana benthamiana* leaves were inoculated with AT12516 carrying pCAMBIA2301 with P_*virB*_::*virE2-TC*. Within 4 to 16 hours after inoculation, leaf tissues were cut and treated with ReAsH reagent without fixation. Three dimensional data sets were acquired in the confocal microscope. We detected punctate red signals in the cytoplasm after detailed review of the recordings and analysis with the 3D objects counter [41] in Fiji software. This tool provides a number of parameters including the integrated intensity and volume of the selected objects. By comparing brightness, size, and location of the spots as seen in the transmitted light images, we were able to distinguish between VirE2-TC protein within the plant cell and whole bacteria containing VirE2-TC. In particular, the whole bacteria were found on the leaf surface, while fluorescent protein puncta were found within the tissue. No such spots were detected in scans of comparable areas of leaf tissue infected with wild-type VirE2 and similarly treated with ReAsH.

**Fig 8:**
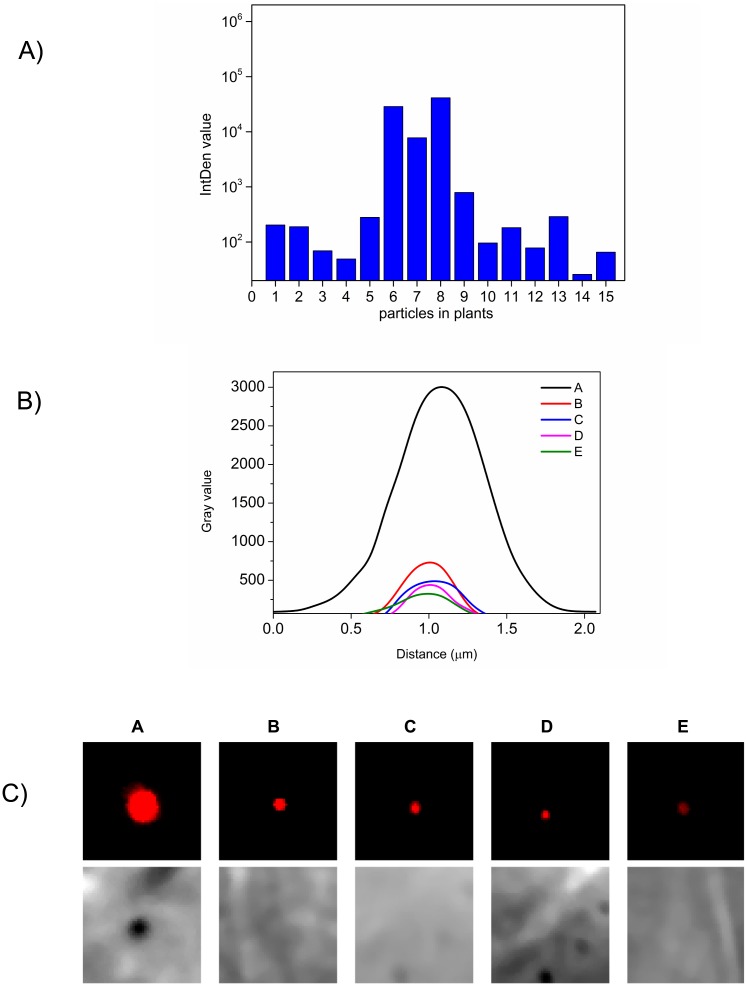
Analysis of ReAsH labeling in leaf tissue to distinguish bacteria from fluorescent VirE2 puncta. (A) Integrated density values of VirE2-TC particles and bacteria labeled with ReAsH reagent. Bars 6 and 8 represent bacteria while the others are VirE2 particles. Bar 7 shows a number of clustered VirE2 particles. Note that the scale is logarithmic in order to display the small particles and bacteria together. (B) Line profiles across a bacterium and several VirE2 particles accentuate the differences in size and total brightness. Note that fluorescent spots of VirE2 are diffraction-limited by the microscope optics. (C) A subset of cropped images showing a bacterium (left) and several VirE2 particles at the same brightness scale. The bacterium image reaches saturation in the figure in order to provide visible contrast in the faint particles. The corresponding region in the differential interference contrast image (acquired simultaneously) is shown below each box. The bacterium appears as a large refractive object while the VirE2 particles are too small to produce a visible signal.

**Fig 9:**
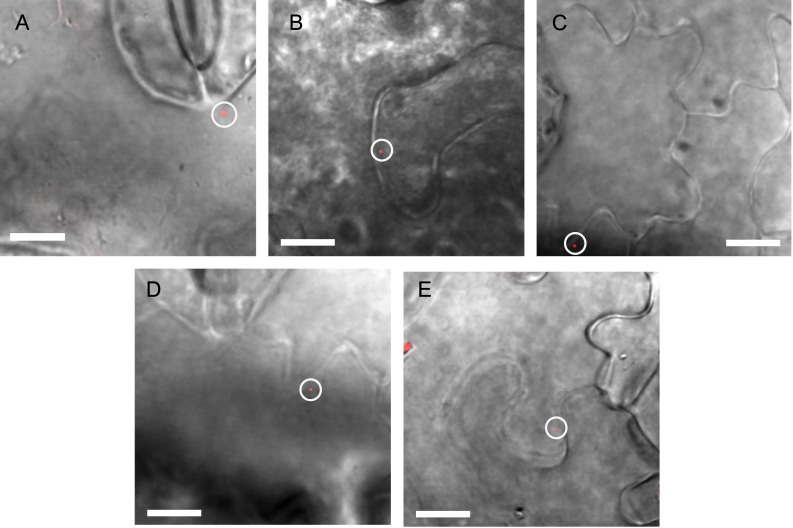
Overlay views of fluorescent puncta in leaf tissue. (A) infiltrated *Agrobacterium* labeled with ReAsH, localized near the upper surface of the *Nicotiana tabacum* leaf. (B-E) VirE2 puncta labeled with ReAsH reagent in *Nicotiana tabacum* (B,E) and *Nicotiana benthamiana* (C,D). Brightness settings are adjusted individually for visibility in the overlays. Scale bar 10 μm.

A small gallery of images with intensity analysis appears in Fig 8. Below each fluorescent panel, the same region is shown by differential interference contrast (DIC). Bacteria are strongly refractive and appear dark, whereas the small puncta originate from an object too small to detect by DIC. Fluorescent signals shown were detected in live cell imaging between 4 and 8 hours post innoculation, which is of relevance to rapid transformation kinetics [28]. Full image overlays showing the particles in cellular context appear in Fig 9. Fluorescent VirE2-TC containing particles were observed only in the cytoplasm, whereas the T-DNA must reach the nucleus in order to effect a transformation.

**Fig 10:**
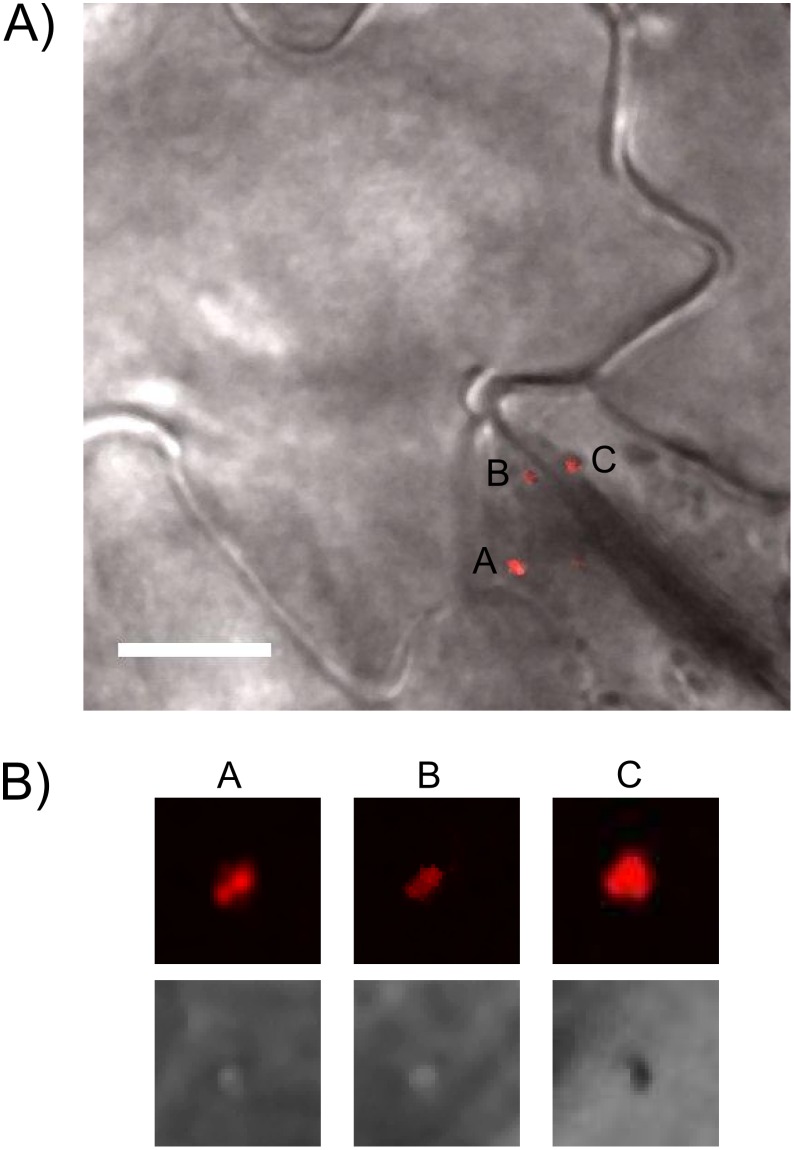
A *virB* mutant strain does not secrete fluorescent puncta. (A) Large, motile fluorescent spots, visible also in the bright field images (B, lower panels), indicate bacteria. Smaller spots representing secreted VirE2 in the host cells were not observed. Scale bar 10 μm.

As an additional negative control, we tested a strain of *Agrobacterium* with the *virB* operon deleted (PC1000). This strain expresses wild-type VirE2 but is unable to secrete it. We confirmed avirulence by transformation with the binary plasmid (pDGB2_alpha2) for expression of YFP in the host; no YFP fluorescence observed after infiltration to *Nicotiana benthamiana* leaves. The strain was then transformed similarly to AT12516 with P_*virB*_::*virE2-TC* in pCAMBIA2301. Fluorescent bacteria were observed in the plant tissue, confirming expression of the VirE2-TC protein (Fig 10), but smaller puncta corresponding to VirE2-TC were not detected in the plant cells.

## Discussion

A number of important roles are attributed to VirE2 during the infection process of *Agrobacterium tumefaciens* [42]. In particular it is believed to function as a capsid by protecting the single strand transfer DNA from degradation in the host cell until it reaches the nucleus. Also notable is that VirE2 synthesized in the plant cell supports efficient transformation by *A*. *tumefaciens virE2-* mutants [11], consistent with a protective role in the cytoplasm.

Recent breakthroughs in imaging have enabled the visualization of bacterial secretion in a growing number of cases. For example the conjugative transfer of DNA between *E*. *coli* was detected specifically via hemi-methylation [43]. Secretion of disease effectors to eukaryotic cells was visualized elegantly with enzymatic amplification of the signal [44]. Bimolecular fluorescence complementation (BiFC) and derivative approaches based on split barrels of GFP-type fluorescent proteins offer a means to detect transferred polypeptides directly [45–47]. Two groups have recently addressed secretion of VirE2 by these means, which enabled real-time tracking of the secreted protein within the host cells [24–26]. A potential pitfall to the GFP complementation approach is that the maturation time to obtain fluorescence reaches several hours [27], by which time the first steps of transformation may already have been completed [48]. A more general issue is that the host plant must be modified to express the split GFP.

In this work we modified VirE2 with a tetracysteine motif that allows direct *in vivo* and *in vitro* labeling using the ReAsH reagent. The related FlAsH reagent was used previously to follow protein secretion by the type 3 secretion system to mammalian cells [30]. One of the advantages of the tetracysteine tags is their small size, as well as the size of the protein-modifying peptide. VirE2 makes N to C terminal “head to tail” contacts in the assembled T-complex, so we chose to insert the tetracysteine peptide within a flexible loop between the major folded domains. Because this loop likely serves as a hinge domain for binding alternative substrates, we tested its interactions carefully in vitro. The modified VirE2-TC bound VirE1 comparably to wtVirE2. Binding to ssDNA was partially impaired but not eliminated. Therefore we expected that this tag may not interfere with function or secretion of the protein and, indeed, VirE2-TC was biologically active in replacing native VirE2 in transformation assays.

A disadvantage of the ReAsH reagent is that it labels cysteine-containing proteins to some degree nonspecifically, which has limited its use for plant studies [49]. Background fluorescence can be reduced by treatment of tissues or cells with a reducing agent before and after labeling. We found 2,3-Dimercaptopropanol (BAL) to be much more effective than the standard 1,2-Ethanedithiol (EDT) reagent used for this purpose. Indeed, only treatment with BAL enabled the detection of punctate spots over background fluorescence in ReAsH-treated plant cells. Detection of fluorescent spots in the cytoplasm was highly challenging by standard fluorescence microscopy and was only possible on review of recorded three dimensional stacks. Previous work in HeLa cells appears to have been considerably more straightforward, so that live imaging was feasible [30].

Based on the *in vitro* data we could estimate roughly what signals might represent VirE2 engaged with a T-strand. Complexes with M13 ssDNA dispersed in a gel typically showed diffraction-limited spots by confocal imaging (Fig 6). This is fully consistent with the size of the circular substrate (7249 bases) in complex with VirE2 [7]. In other words the apparent size of the spot in the image, approximately 300 nm, reflects the fundamental limitations of microscope optics rather than physical size of the complex. Occasionally the DNA clustered to form somewhat larger spots with notably higher intensity, yet they remained smaller and dimmer than ReAsH labeled bacteria (Fig 8). The *Agrobacterium* strain used for secretion of VirE2-TC contains two T-DNA segments: the wild-type on the Ti-plasmid and a shorter one present on the pCAMBIA binary plasmid that encodes the GUS protein. These contain approx. 24,000 [50,51] and 5,400 bases respectively, within a small factor comparable to the M13 model substrate. We can estimate the length of the assembled T-complex from previous work showing 80 bases per 5 nm rise [7], which indicates an upper limit of about 1 μm for the longer T-strand. Given the probability of curling or folding back onto itself, we expect the dimensions of the T-complex to be at or below the resolution limit of the confocal microscope. By comparison with the artificial complexes, the structure-less spots from ReAsH-labeled VirE2 that we observe in the plants match the expected signal for a T-complex both in apparent size and in fluorescence intensity.

In the complex environment of the leaf tissue, detection of diffraction-limited objects (VirE2 particles) demands slow 3D scanning with a dense pixel rate. (In particular, the image of such an object must cover more than a single pixel in order to distinguish it from noise.) In addition to the fluorescence we acquired a DIC image. Using the combination of signals, bacteria are easily distinguished from molecular objects such as VirE2, which do not appear in the DIC channel. On the other hand, we would be unable to detect motion because of the long time required for the three dimensional scan. It is therefore possible that we missed a fraction of the transferred protein or T-complexes during transit through the plant cytoplasm due to the weak signal that would spread over the field of motion.

In the present study it was possible to compare the VirE2 signal *in vivo* to that observed *in vitro* on complexes with defined ssDNA. We consider the major advance to be detection of VirE2 transfer within hours of infection, since transcription of the transgenes initiates on a similar time scale [28]. VirE2 accumulation at longer times might represent excess effector, or priming of the host cells to receive T-DNA from multiple sources. While it has not yet been possible to image the T-strand and VirE2 simultaneously, given the appropriate time scale and the in vitro calibration, the weak, sparse signals detected may represent the secretion relevant to transformation. We expect that future developments in the ReAsH/FlAsH chemistry, in amenable plant model systems, and in the microscopy itself will combine to improve the efficiency of direct substrate detection in the host cell context.
